# Impact of caregiver swimming capability on perceptions of swimming pool supervision of toddlers

**DOI:** 10.1186/s40621-022-00397-3

**Published:** 2022-12-21

**Authors:** Molly B. Johnson, Elizabeth D. Boriack, Carlee M. McConnell, Karla A. Lawson

**Affiliations:** 1 Trauma and Injury Research Center, Dell Children’s Medical Center, 4900 Mueller Blvd, Austin, TX 78723 USA; 2 Kinesiology Department, University of the Incarnate Word, San Antonio, TX 78209 USA; 3 Department of Surgery and Perioperative Care, Dell Medical School, University of Texas at Austin, Austin, TX 78712 USA; 4 Trauma Services, Dell Children’s Medical Center, 4900 Mueller Blvd, Austin, TX 78723 USA

**Keywords:** Swimming, Drowning, Supervision, Caregivers, Parents, Toddlers, Pediatric, Submersion, Attitudes

## Abstract

**Background:**

Drowning is a leading cause of unintentional injury-related death for toddlers within the USA. Keeping toddlers within arm’s reach while swimming is recommended, yet many caregivers do not. Possibly, caregivers’ attitudes are shaped by their expectations about whether they could quickly save a child. The aims of this study are to 1) explore caregivers’ views of arm’s reach pool supervision in various scenarios and 2) understand whether perceptions of arm’s reach pool supervision are impacted by the caregiver’s self-reported capability to swim the length of a standard pool.

**Results:**

Caregivers generally showed agreement with arm’s reach pool supervision; however, arm’s reach supervision was viewed as less necessary when a toddler was in shallow water, wearing a flotation device, or with an older child or teen. There was a significant effect of caregiver swimming capability on perceptions of arm’s reach pool supervision, with non-swimmers and the strongest swimmers showing more positive perceptions of arm’s reach pool supervision than caregivers reporting poor swimming capability. Female caregivers showed significantly more agreement with arm’s reach pool supervision compared with male caregivers. Grandparents and parents showed significantly more agreement with arm’s reach pool supervision than siblings.

**Conclusions:**

Caregivers’ views about what constitutes appropriate supervision are impacted by gender, the relationship to the toddler, and the caregiver’s swimming capability. Findings suggest that a caregiver’s ability to offer close supervision or respond in an emergency may influence their attitudes about what constitutes appropriate supervision. Caregivers may view arm’s reach pool supervision as less necessary when additional layers of protection are in place.

## Background

Drowning is a leading cause of death for toddlers within the USA. In 2018, drowning caused one-third of all accidental fatalities for 1–5 year olds (National Center for Injury Prevention and Control, Centers for Disease Control and Prevention 2022). The impact of drowning is even more wide-spread than submersion fatality data suggests, however. Five times as many children are treated for nonfatal submersion injury than for fatal drowning incidents. Furthermore, submersion injuries have a high morbidity rate, with 50% of submersion injuries requiring hospital admission compared with 6% for other accidental injury categories (National Center for Health Statistics, Centers for Disease Control and Prevention 2022). The high incidence and impact of drowning on young children suggest there is a great need to understand how to reduce the incidence of submersion events, how to make response and rescue efforts more effective, and how to improve outcomes of submersion injuries.

Drowning prevention recommendations by the American Academy of Pediatrics (AAP) highlight the need to institute multiple layers of protection, including the use of home pool security features, such as fencing, pool covers, and alarms; swimming where lifeguards are present; wearing US Coast Guard approved life jackets; training in CPR; developing children’s water competency; and supervising children when they are in or near a body of water. The AAP suggests that, for young non-swimmers and beginner swimmers, supervising adults should be *constantly attentive, in close proximity (i.e., within arm’s reach), and prepared to intervene* (Denny et al. 2019).

Caregiver supervision is one of the most important layers of protection against drowning. However, supervision may not be as *constantly attentive* as the AAP recommends. Research shows that 19–22% of parents of 1–4 year olds in the USA report having left their child at a pool without supervision for more than two minutes (Mackay et al. 2016). Additionally, even though 84% of caregivers report giving their full attention to their toddler when they are at the pool, 38% say they would leave their toddler in the pool to check their phone outside of the pool, and 39% say they would run inside to take a bathroom break while their toddler is in the pool (Johnson et al. 2021). Lapses in supervision can have grave consequences. The majority of fatal drownings for toddlers can be attributed to inadequate supervision (Quan et al. 2011).

Even when a caregiver is not leaving the pool area and is paying consistent attention to their toddler, they may not be *in arm’s reach*. A survey of parents in the USA showed that 40% of parents of 3–4 year olds and 29% of parents of 1–2 year olds do not stay within arm’s reach of their child in the pool (Mackay et al. 2016). There is little research on caregivers’ or parents’ perceptions of arm’s reach pool supervision. The first aim of this study is to understand caregivers’ perceptions of arm’s reach pool supervision, in general, and if these perceptions vary between different swimming scenarios, such as if there is a lifeguard present, or if the toddler is at a pool party (Table 1).

**Table 1:** Percentage of responses to perceptions of arm’s reach pool supervision statements

Statement	Strongly disagree	Disagree	Neutral	Agree	Strongly agree	Mean score*
Parents or caregivers should keep toddlers within arm's reach when they are in the pool	2%	5%	15%	39%	39%	4.07
Toddlers should be kept within arm's reach of their parent or caregiver even if a lifeguard is on duty	1%	5%	19%	43%	32%	4.00
Toddlers should be kept within arm's reach of their parent or caregiver even if they are playing with other toddlers	1%	7%	17%	40%	34%	3.99
Toddlers should be kept within arm's reach of their parent or caregiver even if they are wearing water wings (i.e., arm floaties)	1%	6%	18%	43%	32%	3.97
Toddlers should be kept within arm's reach of their parent or caregiver even if they have had swimming lessons	2%	6%	19%	43%	31%	3.96
Toddlers should be kept within arm's reach of their parent or caregiver even if they are in the pool at a pool party	1%	7%	18%	43%	31%	3.95
Toddlers should be kept within arm's reach of their parent or caregiver even if they are wearing a coast guard approved flotation device (e.g., lifejacket or puddle jumper)	1%	6%	18%	48%	27%	3.93
Toddlers should be kept within arm's reach of their parent or caregiver even if they are in water that is not above the toddler's head	2%	6%	21%	42%	29%	3.91
Toddlers should be kept within arm's reach of a parent or caregiver even if an older child (9–13 years old) is with them	2%	8%	21%	40%	29%	3.87
Toddlers should be kept within arm's reach of a parent or caregiver even if an older child (14–17 years old) is with them	3%	11%	25%	39%	22%	3.67

^*^ Mean score range between 1 = Strongly disagree and 5 = Strongly agree

Prior research shows that caregiver supervision may be impacted by beliefs about a child’s swimming capability. If a child is believed to be able to swim 25 m (m) (the length of a standard pool), their parent is more likely to report an increased sense of safety for the child in open water (Stanley and Moran 2016). Following a course of swimming lessons for their toddlers, parents may believe that they need less close and attentive supervision (Morrongiello et al. 2014). A lessening of supervision as children develop water competency skills supports research that shows that many parents believe children can keep themselves safe from drowning (Morrongiello et al. 2013). Limited research does show that a child’s swimming capability can reduce the risk of having a drowning incident (Brenner et al. 2009), but even toddlers with the highest swimming capability are still at risk. Once a child is actively drowning, the outcome depends on the presence, awareness, and capabilities of other people. Most submersion incidents for children involve a parent or older relative rescuing them from the water (Cody et al. 2004). However, even when a caregiver is supervising attentively, it cannot be assumed that they are *prepared to intervene*. Caregivers may have limited fitness or mobility or may not have the swimming capability necessary to enter deep water and retrieve a child.

According to an American Red Cross survey of a representative sample of US adults from 2014, only 46% reported being able to perform all of the following basic water competency skills: step or jump into the water over your head; return to the surface and float or tread water for one minute; turn around in a full circle and find an exit; swim 25 yards to the exit; and exit from the water without using the ladder. Furthermore, 18% of people who could NOT perform all of the water competency skills expected to supervise a child near the water that summer (American Red Cross 2022). In New Zealand, a survey of parents found that only 45.3% reported that they can swim more than 25 m nonstop (Stanley and Moran 2016). The large proportion of adults who are unable to perform even basic water competency skills may pose a risk if these non-swimmers or poor swimmers are not in *close proximity* when supervising children in the water and are unable to rescue them.

It is possible that caregivers’ attitudes about pool supervision needs are impacted by the caregivers’ water competency. The second aim of this study is to understand how perceptions of arm’s reach pool supervision among caregivers of toddlers are impacted by the caregiver’s self-reported capability to swim the length of a standard pool.

## Results

Results were analyzed for 650 participants. Eighty percent of the participants in the survey were parents (parents/foster parents/step-parents) of a toddler. Sixty-two percent were male. Fifty-four percent were 25–34 yrs old. Seventy-one percent were white. Fifty-one percent had an income of $50,000–$99,999; 30% had an income of $25,000–$49,999; 12% had an income of $100,000 or more; and 7% had an income less than $25,000. Sixty-three percent had a bachelor’s degree; 18% had an advanced degree; 13% had some college; 6% had a high school diploma or less. There was at least one participant from every state in the USA except Alaska and South Dakota. See Table 2 for detailed demographic information.

**Table 2:** Characteristics of survey participants and perceptions of arm’s reach pool supervision mean scores and standard deviations

	Category	*n*	%	Mean	SD
Swimming capability*	Yes, easily (a)	395	60.8	40.04	6.98
	Yes, but it would be hard (b)	208	32.0	37.82	6.38
	Probably not (b)	28	4.3	37.29	9.06
	Definitely not (a)	19	2.9	43.89	6.15
Caregiver Relationship*	Parent (a)	520	80.0	39.51	7.24
	Grandparent (a)	58	8.9	40.45	5.40
	Aunt/uncle/cousin (ab)	44	6.8	38.09	5.56
	Sibling (b)	28	4.3	35.50	5.95
Gender*	Female	248	38.2	40.33	6.95
	Male	402	62.1	38.71	6.95
Age	18–24 yrs	23	3.5	38.74	5.86
	25–34 yrs	352	54.2	38.92	6.89
	35–44 yrs	200	30.8	40.22	7.46
	45–54 yrs	47	7.2	39.17	6.73
	55 yrs or older	28	4.3	38.71	5.83
Race/ethnicity	White	461	70.9	39.13	6.76
	Black	118	18.2	38.92	7.78
	Asian	18	2.8	42.06	6.26
	Mixed race	22	3.4	41.64	6.13
	Hispanic/Latino	31	4.8	40.52	7.77
All caregivers		650	100	39.32	6.99

^*^ indicates variables with significant differences in perceptions of arm’s reach pool supervision. Categories followed by the same letter are not significantly different using Tukey’s HSD. Swimming capability question: Can you swim 25 m (80 feet, the length of a standard pool) without touching the bottom?

### Swimming capability

For self-reported swimming capability (Table 2), 60% answered *Yes, easily* to being able to swim 25 m without touching the bottom; 32% answered *Yes, but it would be hard*; 4% answered *Probably not*; and 3% answered *Definitely not*.

### Perceptions of arm’s reach pool supervision

The average perceptions of arm’s reach pool supervision score was consistent with agreement that caregivers should keep toddlers within arm’s reach in a pool in various situations (mean 39.32; SD: 6.99). The statement that elicited the highest agreement was *Parents or caregivers should keep toddlers within arm's reach when they are in the pool*, with 78% strongly agreeing or agreeing. The statement that elicited the lowest agreement was *Toddlers should be kept within arm's reach of a parent or caregiver even if an older child (14–17 years old) is with them,* with 61% strongly agreeing or agreeing. Between 6 and 14% of caregivers disagreed or strongly disagreed with each statement. See Table 1 for greater detail on agreement with individual statements.

### Impacts on perceptions of arm’s reach pool supervision

There was a significant effect of caregiver swimming capability on perceptions of arm’s reach pool supervision (*p* = 0.0002). Caregivers who could *Definitely not* swim 25 m showed most agreement with arm’s reach supervision (mean 43.89; SD 6.15), followed by those who answered, *Yes, easily* (mean 40.04; SD: 6.98). The lowest agreement was shown by caregivers who answered, *Yes, but it would be hard* (mean 37.82; SD 6.38) and *Probably not* (mean 37.29; SD 9.06). There were significant differences in perceptions of arm’s reach pool supervision based on the caregiver relationship (*p* = 0.0082), with grandparents (mean 40.45; SD 5.40), parents (mean 39.51; SD 7.24), and aunts/uncles/cousins (mean 38.09; SD 5.56) showing the most positive perceptions. Siblings (mean 35.50; SD 5.95) showed perceptions of arm’s reach pool supervision that were significantly lower than parents and grandparents. There were significant differences in perceptions of arm’s reach pool supervision based on gender (*p* = 0.0027), with female caregivers showing higher agreement (mean 40.33; SD 6.95) compared with male caregivers (mean 38.71; SD 6.95). There were not significant differences in perceptions of arm’s reach pool supervision based on age (*p* = 0.765) or race (*p* = 0.165). See Table 2 for means and standard deviations for all analyses.

## Discussion

This study showed that, although there was general agreement with arm’s reach pool supervision, caregivers of toddlers perceived arm’s reach pool supervision to be less necessary when there are additional layers of protection in place, like if the toddler is wearing a flotation device, swimming in shallow water, or being watched by an older child or teen. Additionally, results highlight that the caregiver’s self-reported swimming capability to swim the length of a standard pool impacted their perceptions, with poor swimmers showing less support for arm’s reach pool supervision than good swimmers and non-swimmers.

This study demonstrated that caregivers generally support the need for arm’s reach pool supervision, with the average perceptions of arm’s reach pool supervision score showing agreement, and with over 60% of respondents agreeing or strongly agreeing with each statement about arm’s reach supervision. However, results also showed that support for arm’s reach pool supervision may depend on situational factors. Despite 39% of respondents strongly agreeing with a general statement that caregivers should keep toddlers within arm’s reach while in the pool, when asked about scenarios where there is a another child or teen with the toddler, the toddler is in a lifejacket, or the toddler is in water that is not above their head, fewer than 30% of respondents strongly agreed (Table 1). It is not uncommon for toddlers to drown in seemingly safer situations, like being in shallow water or watched by older kids, (Quan et al. 2011) so it is concerning that caregivers would be willing to reduce the closeness of their supervision in these scenarios. Drowning prevention requires multiple layers of protection. Drowning prevention messaging should highlight how adding a layer of protection, like using a lifejacket or taking swimming lessons, does not mean that other layers can be lessened and that attentive, close supervision is a critical layer of protection for all swimming scenarios.

This study showed that perceptions of arm’s reach pool supervision are impacted by the relationship the caregiver has to the child, with siblings showing less agreement with arm’s reach pool supervision compared with grandparents and parents. This cannot be attributed to age, though, as there was not a significant difference in perceptions found based on age. Our lack of a significant finding based on age is in contrast, however, with actual observations of caregiver supervision behavior, which show better supervision by younger adult caregivers (Petrass and Blitvich 2012). In this study, female caregivers showed more agreement with arm’s reach pool supervision than male caregivers. This is consistent with research showing that male caregivers see themselves as playing a less essential role in reducing drowning risk around water (Moran 2009). No significant differences in perceptions of arm’s reach pool supervision were found based on race in this study. However, a higher drowning burden in the USA for racial minorities indicates a need to address potential health disparities in drowning prevention efforts (Saluja et al. 2006).

The results of this study suggest that caregivers’ sense of whether they possess the swimming capability to save a drowning child impacts how close they believe a caregiver needs to be to a toddler when they are swimming. As might be expected, non-swimmers showed the highest support for keeping toddlers within arm’s reach when they are in a swimming pool. Within this group of non-swimmers, it is likely there are some who are scared of water. According to a survey by the American Red Cross, 30% of adult non-swimmers report being scared of the water (American Red Cross 2022). Potentially fear or discomfort works in toddlers’ favor, leading caregivers to have closer, more attentive supervision when they are not comfortable in the water or not capable of swimming.

Swimmers who are confident that they could swim 25 m without touching the bottom showed agreement with arm’s reach pool supervision. It could be predicted that adults who are most comfortable with their own swimming capability would be the most lax with supervision, but this is opposite of what we found. It is possible that capable swimmers have enough experience with swimming to be aware of how challenging it would be to swim across a pool to retrieve a drowning child. This understanding of what it might take to rescue a child might lead confident swimmers to value the need for arm’s reach supervision.

Though still in agreement with arm’s reach pool supervision, caregivers who responded *Yes, but it would be hard* and *Probably not* to whether they could swim 25 m without touching the bottom of the pool showed perceptions approaching a neutral attitude toward arm’s reach pool supervision. These poor swimmers comprised more than 1/3 of caregivers surveyed and pose a problematic situation for pool supervision. Poor swimmers' support for arm’s reach pool supervision is not as high as ideal, yet they don’t have the swimming capability that would likely be needed to save a drowning child that was not in arm’s reach. These findings suggest that a caregivers’ own experience and capability related to swimming impacts decisions they may make about supervision needs. It also highlights that it might be necessary to put in extra effort to reach caregivers who are poor swimmers since they have neither the experience of confident swimmers nor the fear or discomfort of non-swimmers that might drive these other groups to offer a higher level of drowning protection to the children they care for.

This study found that 39.2% of caregivers were NOT confident they could swim 25 m. Prior research on swimming capability of adults in the USA has shown similar results. The Red Cross reports that 35% of adults in the USA say they can NOT swim 25 m (American Red Cross 2022). These data show that a high proportion of adults cannot swim even one length of a standard pool. This means that more than one-third of adults who may be supervising a child in the water are not *prepared to intervene* if that child was drowning.

The high percentage of caregivers who cannot easily swim 25 m in addition to the lower support for arm’s reach supervision among poor swimmers suggests that it is not just children that need swimming lessons. Drowning prevention recommendations should include suggestions for caregivers to improve their own water competency in order to be more prepared for supervising children in or around bodies of water. Additionally, it could be suggested that parents, when designating a supervisor for children in the water, should not just ask whether that supervisor is capable of *watching* the child but whether that supervisor is capable of *rescuing* a child in need. This research supports the AAP recommendation that supervisors of beginning or non-swimmers should be *constantly attentive, in close proximity (i.e., within arm’s reach), and prepared to intervene* (Denny et al. 2019).

There are limitations to the conclusions that can be drawn from our findings. Although a number of variables were statistically significant, because the survey was only validated statistically, it cannot be assumed that the differences are functionally or clinically significant, in practice. Additionally, some variables’ standard deviations overlapped, suggesting more overlap between scores than statistical differences might suggest.

This study provides insightful data on swimming capability, but the information received was all self-report. Research shows that self-reported swimming capability and actual swimming capability are not always matched (Petrass et al. 2012). Additionally, perceptions of arm’s reach pool supervision asked generally about the need for arm’s reach supervision for toddlers in different swimming pool scenarios, not about the caregiver’s actual supervision behavior. Research previously published on this same group of participants showed that the perceptions of arm’s reach pool supervision scale we used in this paper is predictive of self-reported supervision behavior of the caregiver with their own toddler (Johnson et al. 2021). However, these self-reported perceptions and behaviors may not reflect the actual behavior adopted by these caregivers when they are supervising the toddler they care for. More observational research is needed to understand actual supervision behavior. Future research should also explore child drowning rescue attempts to better understand what scenarios or decisions could add unnecessary seconds or minutes to the rescue time, delaying rescue attempts. Research could highlight how often a caregiver asks a bystander to retrieve the child or leaves the scene to get someone else to rescue the child. Research on supervision around bodies of water and on actual drowning incidents can help highlight how caregivers can be better prepared to intervene in a drowning situation. Additionally, a better understanding of the risks posed to people attempting to rescue a drowning person are needed in order to keep caregivers and bystanders from undue risk to their own lives when someone else is drowning.

## Conclusions

This study demonstrated general agreement with arm’s reach pool supervision by caregivers of toddlers, but that caregivers may believe arm’s reach supervision is less necessary when other layers of protection are in place, like flotation devices or when older children are with the toddler. These findings highlight the need for educational training and water safety media campaigns that emphasize the importance of arm’s reach supervision for toddlers in and around bodies of water. This study also showed that perceptions of arm’s reach pool supervision are impacted by the swimming capability of the caregiver, with confident swimmers and non-swimmers showing the most positive support for arm’s reach pool supervision. The lower support for arm’s reach pool supervision by poor swimmers and the high percentage of caregivers who are poor and non-swimmers suggest the need for water competency and swimming skill training for adult caregivers.

## Methods

### Survey procedures

An anonymous survey of caregivers of toddlers was conducted. Participants were recruited from across the USA using the online Amazon MTurk platform using the following headline: Parents or caregivers of 1–4 yr olds—water safety survey. Participants were only allowed to navigate to the survey if they answered that they were over 18 years of age and that they were the caregiver of a toddler (1–4 years old). The study was approved by the University of Texas at Austin Health Sciences Institutional Review Board. Participants reviewed and agreed to an informed consent document before they started the survey.

The survey asked about the demographics and background of the caregiver and toddler and rated agreement with statements about perceptions of arm’s reach pool supervision for toddlers. To assess reported swimming capability, participants were asked, *Can you swim 25 m (80 feet, the length of a standard pool) without touching the bottom?* To ensure high quality data from the MTurk workers, a score was created that summed a number of poor data quality indicators, such as a completion time of less than 5 min, odd textbox entry answers, and contradictory answers on multiple choice questions. Of 916 completed surveys, 266 surveys with more than 2 low quality indicators were not included in analyses.

### Perceptions of arm’s reach pool supervision scoring

Caregivers were asked to rate their attitude about arm’s reach pool supervision for toddlers using a 5-point Likert scale from strongly disagree to strongly agree on ten statements that included various scenarios (see Table 1). All statements were worded in a parallel manner so that agreement reflected that caregivers should keep toddlers within arm’s reach in each scenario. The ten statement scores were summed to create a single perceptions of arm’s reach pool supervision score that had a possible range of 10–50 where 10 reflected strong disagreement with arm’s reach supervision in all given scenarios and 50 reflected strong agreement with arm’s reach supervision in all given swimming pool scenarios.

Before analyzing the results, an item analysis was performed on the perceptions of arm’s reach pool supervision statements. Pearson correlations were used to assess whether each statement was consistent with the other statements. Perceptions of arm’s reach pool supervision statements showed moderate positive correlations for all pairs of statements (*ρ* = 0.30–0.66). No one statement received low correlations with all other statements, indicating that no statement was inconsistent with the rest.

In order to assess the validity of the perceptions of arm’s reach pool supervision score, an ANOVA determined differences in mean perceptions scores between the *yes* and *no* answer groups to the survey question, *Do you think it is necessary to keep toddlers within arm’s reach when they are in the pool regardless of the situation?* A significantly lower score for those who answered *no* (mean 34.70; SD 7.63) compared to those who answered *yes* (mean 39.68; SD 6.82) supported the validity of the perceptions of arm’s reach pool supervision score (*F*(1,648) = 22.44, *p* = 0.0000). As expected, the results demonstrate less agreement with arm’s reach pool supervision in various scenarios for those who believe it is not necessary.

### Data analysis

Scoring of the perceptions of arm’s reach pool supervision section was performed in Excel. All other statistical analyses were conducted using STATA (version 12.1, STATA, inc, College Station, TX). Univariate analyses of variance were used to determine differences in mean perceptions of arm’s reach pool supervision scores based on swimming capability, gender, caregiver relationship, age, and race. Tukey’s HSD post hoc tests were used to determine significant differences in perceptions of arm’s reach pool supervision scores between swimming capability categories and caregiver relationship categories. Significance was defined as *p* < 0.049, using a Bonferroni adjustment for multiple univariate analyses.

## Data Availability

We do not have the consent of the participants to make data public.

## Abbreviations

MTurk: Amazon mechanical turk
AAP: American academy of pediatrics
ANOVA: Analysis of variance
m: Meters
parents: Parents/foster parents/step-parents
SD: Standard deviation
Tukey’s HSD: Tukey’s honestly significant difference
US: United States

## References

[CR13] American Red Cross. Water safety poll [cited 2022 Jan 20; https://www.redcross.org/content/dam/redcross/enterprise-assets/pdfs/Water-Safety-Poll-2014.pdf]

[CR1] Brenner RA, Taneja GS, Haynie DL, Trumble AC, Qian C, Klinger RM, Klebanoff MA (2009). Association between swimming lessons and drowning in childhood: a case-control study. Arch Pediatr Adolesc Med.

[CR2] Cody BE, Quraishi AY, Dastur MC, Mickalide AD (2004). Clear danger: a national study of childhood drowning and related attitudes and behaviors.

[CR3] Denny SA, Quan L, Gilchrist J, McCallin T, Shenoi R, Yusuf S, Hoffman B, Weiss J (2019). Council on injury, violence, and poison prevention: prevention of drowning. Pediatrics.

[CR4] Johnson MB, Boriack ED, McConnell CM, Williams SR, Naiditch JA, Lawson KA (2021). Predictors of swimming pool supervision for caregivers of toddlers. J Inj Violence Res.

[CR5] Mackay JM (2016). Keeping kids safe in and around water: exploring misconceptions that lead to drowning.

[CR6] Moran K (2009). Parent/caregiver perceptions and practice of child water safety at the beach. Int J Inj Contr Saf Promot.

[CR7] Morrongiello BA, Sandomierski M, Schwebel DC, Hagel B (2013). Are parents just treading water? The impact of participation in swim lessons on parents’ judgments of children's drowning risk, swimming ability, and supervision needs. Accid Anal Prev.

[CR8] Morrongiello BA, Sandomierski M, Spence JR (2014). Changes over swim lessons in parents’ perceptions of children’s supervision needs in drowning risk situations: “His swimming has improved so now he can keep himself safe”. Health Psychol.

[CR12] National Center for Injury Prevention and Control, Centers for Disease Control and Prevention. Web-based injury statistics query and reporting system [cited 2022 Jan 20; www.cdc.gov/injury/wisqars]

[CR16] National Center for Health Statistics, Centers for Disease Control and Prevention. Wide-ranging online data for epidemiologic research [cited 2022 Jan 20; http://wonder.cdc.gov]

[CR9] Petrass LA, Blitvich JD (2012). The nature of caregiver supervision of young children in public pools. Int J Aquat Res Educ.

[CR14] Petrass LA, Blitvich JD, Keith McElroy G, Harvey J, Moran K. Can you swim? self-report and actual swimming competence among young adults in Ballarat Australia. Int J Aquat Res Educ 2012, 10.25035/ijare.06.02.05.

[CR10] Quan L, Pilkey D, Gomez A, Bennett E (2011). Analysis of paediatric drowning deaths in Washington State using the child death review (CDR) for surveillance: what CDR does and does not tell us about lethal drowning injury. Inj Prev.

[CR11] Saluja G, Brenner RA, Trumble AC, Smith GS, Schroeder T, Cox C (2006). Swimming pool drownings among US residents aged 5–24 years: understanding racial/ethnic disparities. Am J Public Health.

[CR15] Stanley T, Moran K. Parental perceptions of water competence and drowning risk for themselves and their children in an open water environment. Int J Aquat Res Educ 2016, 10.25035/ijare.10.01.04.

